# Factors Associated with Methicillin-Resistant *Staphylococcus aureus* Infection Among Patients with Sepsis Presenting to Emergency Departments: A Nationwide Multicenter Cohort Study

**DOI:** 10.3390/antibiotics15090915

**Published:** 2026-09-16

**Authors:** Hyeji Lee, Dong-Gon Hyun, Chae-Man Lim, Ryoung-Eun Ko, Gee Young Suh, Won Young Kim

**Affiliations:** 1 Department of Emergency Medicine, College of Medicine, Chungnam National University, 266 Munwha-ro, Jung-gu, Daejeon 35015, Republic of Korea; hyejiworks@naver.com; 2 Department of Pulmonary and Critical Care Medicine, Asan Medical Center, University of Ulsan College of Medicine, 88 Olympic-ro 43-gil, Songpa-gu, Seoul 05505, Republic of Korea; dghyun@amc.seoul.kr (D.-G.H.); cmlim@amc.seoul.kr (C.-M.L.); 3 Department of Critical Care Medicine, Division of Pulmonary and Critical Care Medicine, Department of Medicine, Samsung Medical Center, Sungkyunkwan University School of Medicine, 81 Ilwon-ro, Gangnam-gu, Seoul 06351, Republic of Korea; koryoungeun@gmail.com (R.-E.K.); smccritcare@gmail.com (G.Y.S.); 4 Department of Emergency Medicine, Asan Medical Center, University of Ulsan College of Medicine, 88 Olympic-ro 43-gil, Songpa-gu, Seoul 05505, Republic of Korea

**Keywords:** sepsis, methicillin-resistant *Staphylococcus aureus*, antimicrobial stewardship, risk factors, prevalence, emergency medicine

## Abstract

**Background/Objectives**: Prompt initiation of appropriate antibiotics is essential for sepsis management. While early anti-methicillin-resistant *Staphylococcus aureus* (MRSA) therapy benefits confirmed cases, unnecessary use in low-risk patients promotes antimicrobial resistance. Current guidelines recommend risk-based empiric coverage, but specific predictors in the emergency department (ED) remain poorly defined. Therefore, this study aims to identify clinical factors independently associated with MRSA infection in ED patients with sepsis. **Methods**: This retrospective analysis used data from a prospective, nationwide, multicenter cohort in the Korean Sepsis Alliance registry. The study included 11,477 adults with sepsis from 16 tertiary or university-affiliated EDs between September 2019 and December 2022. Factors independently associated with MRSA infection were identified with multivariable logistic regression. **Results**: MRSA infection occurred in 189 of 11,477 patients (1.65%; 95% confidence interval [CI], 1.43–1.90%), representing 2.86% (95% CI, 2.49–3.29%) of patients with a microbiologically identified causative pathogen. Catheter-related infection showed the strongest association (odds ratio [OR], 5.98; 95% CI, 1.93–18.49). Other factors independently associated with MRSA included hospitalization within 90 days (OR, 1.65; 95% CI, 1.14–2.39) and elevated C-reactive protein ≥ 6 mg/dL (data-derived cutoff; OR, 2.18; 95% CI, 1.43–3.38). Conversely, abdominal (OR, 0.19; 95% CI, 0.09–0.43) and urinary (OR, 0.40; 95% CI, 0.21–0.77) sources were linked to significantly lower risk. **Conclusions**: MRSA infection was uncommon (1.65%) among ED patients with sepsis. Catheter-related infection was most strongly associated with MRSA, whereas abdominal and urinary sources were associated with lower odds. These findings may help inform risk stratification and support antimicrobial stewardship in the ED.

## 1. Introduction

Timely initiation of appropriate antibiotic therapy is essential for sepsis management, particularly in the emergency department (ED), where clinical decisions must often be made before culture results or prior infection history are available. *Staphylococcus aureus* is among the leading causes of bacterial infection-related mortality worldwide, having been associated with an estimated 1,105,000 deaths globally in 2019 [1]. Methicillin-resistant *Staphylococcus aureus* (MRSA) infection also carries significantly higher mortality than that of methicillin-susceptible *S. aureus* infection (1-month mortality, 22.7% vs. 10.6%) [2]. Delayed active therapy may therefore worsen outcomes [3,4]. However, unnecessary anti-MRSA therapy may increase the risk of nephrotoxicity, treatment burden, and antimicrobial resistance [5]. Thus, identifying factors associated with MRSA infection may help inform risk stratification and antimicrobial stewardship efforts in the ED.

The current Surviving Sepsis Campaign guidelines emphasize the need for improved clinical prediction tools and rapid diagnostics to inform decisions regarding MRSA coverage in empiric regimens [3]. Reported MRSA risk factors include previous MRSA infection or colonization, recent intravenous antibiotic use or hospitalization, recurrent skin and soft tissue infections (SSTIs), chronic wounds, invasive devices, hemodialysis, and greater illness severity [6]. However, most supporting evidence comes from selected populations, such as patients with pneumonia, bloodstream infections, intensive care unit (ICU) admission, or healthcare-associated infections [7,8,9]. These findings may not be generalizable to the heterogeneous population of patients presenting to the ED with sepsis. Moreover, substantial international variation exists in the management of *S. aureus* bacteremia, with no international consensus on the standard of care [10], and regional differences in pathogen distribution suggest that region-specific, rather than uniform empirical antibiotic recommendations may be more appropriate [1]. Recent ED data also suggest that MRSA infection is uncommon despite frequent empiric vancomycin use and that traditional risk factors do not consistently identify culture-confirmed MRSA infection. In particular, the contribution of the suspected infection source to MRSA risk remains insufficiently characterized.

Therefore, using a prospective, nationwide, multicenter sepsis registry, this study aims to estimate the prevalence of clinically significant MRSA infection and identify clinical factors independently associated with MRSA infection among adults presenting with sepsis to Korean EDs. We evaluated readily available clinical factors, including healthcare exposures, comorbidities, illness severity, laboratory findings, and suspected infection sources.

## 2. Results

**Figure 1:**
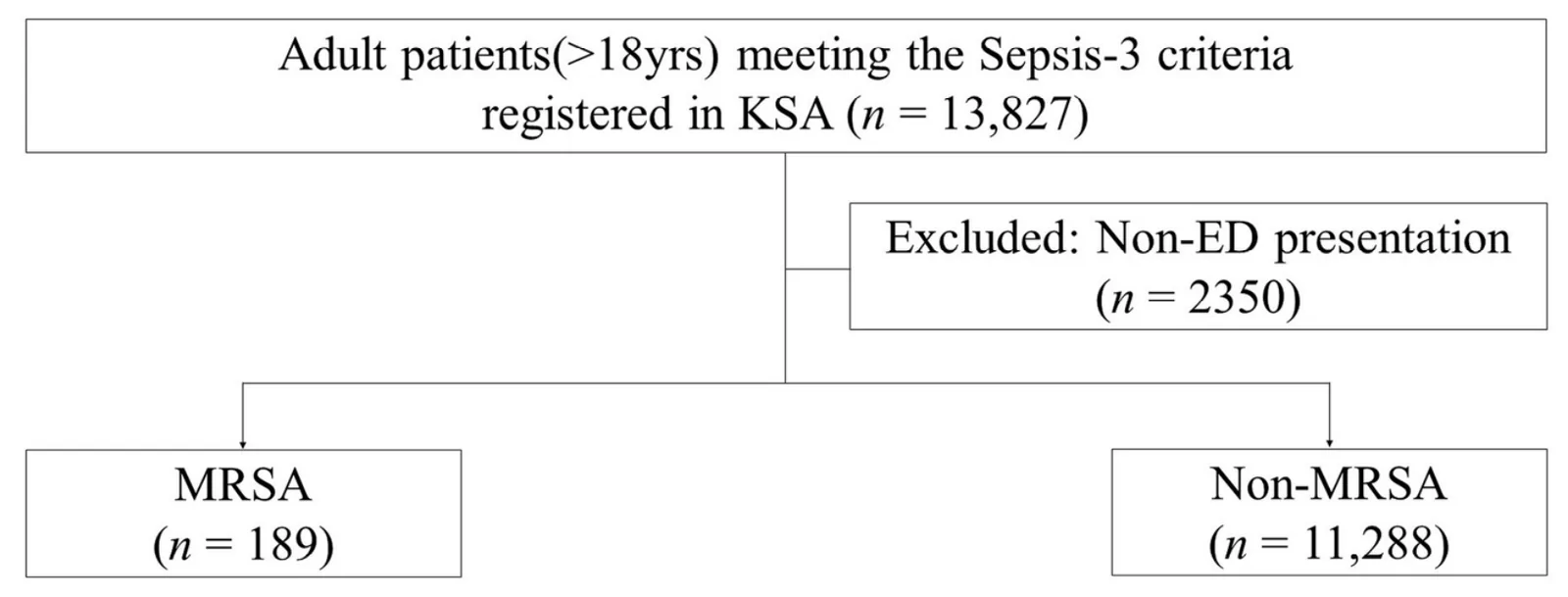
Flowchart of the study population selection. KSA, Korean Sepsis Alliance; ED, emergency department; MRSA, methicillin-resistant *Staphylococcus aureus*.

Among 13,827 adult patients with sepsis registered in the Korean Sepsis Alliance registry, 11,477 patients who presented to the ED were included in the final analysis (Figure 1). Pathogens were identified in 6606 patients (57.6%), and MRSA was isolated in 189 patients (1.65%).

### 2.1. Baseline Characteristics

Baseline characteristics differed between patients with and without MRSA infection (Table 1). Patients with MRSA were older (mean age, 75.9 vs. 73.1 years; *p* = 0.005) and had higher Clinical Frailty Scores (median, 7 [5–8] vs. 6 [4–7]; *p* < 0.001), whereas Charlson Comorbidity Index scores were similar (median, 7 [5–8] vs. 6 [5–8]; *p* = 0.168). Patients with MRSA had higher initial Sequential Organ Failure Assessment (SOFA) scores (median, 7 [5–9] vs. 6 [4–8]; *p* = 0.001), although the septic shock incidence did not differ (15.9% vs. 14.4%; *p* = 0.637). Chronic kidney (16.9% vs. 10.2%; *p* = 0.004) and chronic neurologic diseases (53.4% vs. 39.0%; *p* < 0.001) were more common among patients with MRSA.

Table 2 presents infection-related variables and healthcare-associated factors. Pulmonary infections (54.5% vs. 41.4%), SSTIs (5.3% vs. 2.2%), and catheter-related infections (2.6% vs. 0.3%) were more frequent among patients with MRSA infection. Conversely, abdominal (4.8% vs. 22.2%) and urinary infections (8.5% vs. 15.8%) were less common. Community-acquired infections were less common among patients with MRSA (47.1% vs. 69.7%; *p* < 0.001), whereas nursing home-acquired (17.5% vs. 10.3%; *p* = 0.002) and nursing hospital-acquired infections (28.6% vs. 14.3%; *p* < 0.001) were more frequent. Hospitalization within the past 90 days was also more common among patients with MRSA infection (60.3% vs. 44.9%; *p* < 0.001). Although numerical differences were observed in recent antimicrobial use (37.0% vs. 31.1%; *p* = 0.094), dialysis (6.3% vs. 3.5%; *p* = 0.053), and wound care (4.8% vs. 2.7%; *p* = 0.137), none of these differences reached statistical significance.

### 2.2. Laboratory Findings

Laboratory data differed between groups (Table 3). Neutropenia rates and white blood cell counts were similar between groups. Patients with MRSA had lower hemoglobin levels (10.3 vs. 10.9 g/dL; *p* = 0.003) and higher platelet counts (211.9 vs. 192.5 × 10^3^/µL; *p* = 0.028). Additionally, patients with MRSA had higher blood urea nitrogen (46.9 vs. 39.8 mg/dL; *p* = 0.001), lower serum albumin (2.7 vs. 2.9 g/dL; *p* < 0.001), and higher C-reactive protein (CRP) levels (15.9 vs. 13.1 mg/dL; *p* < 0.001). Chloride concentrations were also slightly higher in patients with MRSA (103.3 vs. 101.8 mmol/L; *p* = 0.021). Sodium and PaCO_2_ levels showed nonsignificant trends toward higher values in patients with MRSA.

### 2.3. Factors Associated with Methicillin-Resistant Staphylococcus aureus Infection

Multivariable logistic regression revealed several factors independently associated with MRSA infection (Table 4). Of the 11,477 patients, 10,288 were included in the multivariable analysis after excluding those with missing values for covariates included in the final regression model. Higher CRP levels were associated with increased odds of MRSA infection as a continuous variable (odds ratio [OR] = 1.020 per 1-mg/dL increase; 95% confidence interval [CI], 1.006–1.035; *p* = 0.006). We also examined a data-derived cutoff of ≥6 mg/dL as an exploratory threshold rather than a prospectively validated treatment-decision cutoff. At this cutoff, CRP was similarly associated with increased odds of MRSA infection (OR = 2.187; 95% CI, 1.413–3.386; *p* < 0.001). CRP alone showed modest discrimination for MRSA infection (area under the receiver operating characteristic curve [AUROC] = 0.588; 95% CI, 0.548–0.628), with a sensitivity, specificity, positive predictive value (PPV), and negative predictive value (NPV) of 84.4%, 31.5%, 2.0%, and 99.2% at this cutoff, respectively. Optimism-corrected bootstrap estimates were similar (AUROC = 0.588; sensitivity, 82.4%; specificity, 31.5%; PPV, 1.9%; NPV, 99.1%). Hospitalization within the previous 90 days was also associated with increased odds of MRSA infection (OR = 1.655; 95% CI, 1.144–2.393; *p* = 0.007). Among the infection sources, catheter-related infections were strongly associated with MRSA infection (OR = 5.988; 95% CI, 1.939–18.491; *p* = 0.002), while abdominal (OR = 0.199; 95% CI, 0.092–0.430; *p* < 0.001) and urinary infections (OR = 0.407; 95% CI, 0.215–0.773; *p* = 0.006) were associated with lower odds of MRSA infection. In a sensitivity analysis using Firth’s penalized likelihood logistic regression to address potential sparse-data bias associated with catheter-related infection, the association remained directionally consistent and statistically significant (OR = 6.322; 95% CI, 2.147–18.613; *p* < 0.001), compared with an OR of 5.988 (95% CI, 1.939–18.491) in the conventional model.

## 3. Discussion

In this nationwide cohort of 11,477 patients with sepsis presenting to the ED, the prevalence of MRSA infection was low, accounting for 1.6% of cases. Multivariable analysis revealed three factors independently associated with MRSA infection: hospitalization within the previous 90 days, elevated CRP levels (≥6 mg/dL), and catheter-related infections. In contrast, abdominal and urinary tract infections (UTIs) were associated with lower odds of MRSA infection, whereas traditional risk factors, including hemodialysis, immunosuppression, and recent antibiotic exposure, were not significantly linked to MRSA infection. These findings may improve risk stratification for empiric MRSA coverage in the ED, where treatment decisions often rely on limited clinical information.

In our cohort, the overall prevalence of MRSA infection among ED patients with sepsis was only 1.6%, well below the 5% threshold that a recent review identified as a reasonable cutoff for favoring β-lactam therapy over empiric anti-MRSA coverage [11]. Accordingly, the relevant clinical challenge in this setting is not whether MRSA should always or never be covered empirically, but how to identify patients in whom the probability of MRSA is sufficiently high to justify early empiric coverage.

Catheter-related infection showed the strongest association with MRSA infection, consistent with previous findings highlighting the role of invasive devices in *S. aureus* bacteremia [12,13]. This finding is biologically plausible because *S. aureus* readily adheres to intravascular devices and forms biofilms that reduce antibiotic susceptibility and facilitate immune evasion. Similarly, delayed catheter removal is associated with a higher rate of bacteremia relapse (12.7% vs. 4.7%) [11]. However, because catheter-related source classification in our registry could incorporate culture results obtained after ED disposition, this association may be most relevant once catheter-related infection is clinically suspected rather than as a basis for MRSA coverage decisions made at ED arrival.

*S. aureus* is an uncommon primary urinary pathogen, and its isolation from urine may reflect urinary instrumentation, colonization, or hematogenous dissemination. The inverse association between urinary source and MRSA in our cohort should therefore be interpreted in the context of the low overall prevalence of MRSA urinary infection and not generalized to patients with specific urinary devices or prior MRSA infection. The predominantly community-onset presentation of sepsis cases in the ED may explain the lower odds of MRSA infection in UTI-associated sepsis [14,15,16].

Intra-abdominal infections encompass a broad spectrum of conditions involving microbial contamination of the abdominal cavity [17]. The predominant pathogens vary based on infection site and generally shift from aerobic to anaerobic organisms along the gastrointestinal tract. Common causative bacteria include *Escherichia coli* (23.8%), *Enterococcus* species (23.1%), and *Klebsiella* species (19.8%). MRSA is an uncommon cause of intra-abdominal infections, although older patients and those with significant comorbidities, prior hospitalization or surgery, recent antibiotic exposure, or known MRSA colonization are considered to be at higher risk [18,19]. These findings are consistent with our finding that abdominal infection sources are associated with lower odds of MRSA infection.

Although SSTIs are traditionally considered common sources of MRSA infection, particularly in regions with a high prevalence of community-associated MRSA, including the USA300 clone, they were not independently associated with MRSA infection in our cohort [20]. However, these findings align with those of European studies reporting lower MRSA virulence and prevalence in skin infections [21,22]. These regional differences underscore the importance of incorporating local microbiological data, including strain distribution and resistance patterns, into empiric antibiotic therapy rather than relying solely on generalized historical models.

Respiratory specimens (sputum or endotracheal aspirate) were the second most common source of MRSA isolation in our cohort (57/189, 30.2%), after blood (50.8%). Pulmonary infection was the most common infection source overall in the cohort (4774/11,477, 41.6%). Considered alone, pulmonary source was associated with increased odds of MRSA (crude OR = 1.74; 95% CI, 1.28–2.37). However, this association was no longer significant in the multivariate model (OR = 0.989; 95% CI, 0.671–1.457; *p* = 0.955; Table 4). This discrepancy likely reflects a comparison artifact. Compared to a heterogeneous reference group comprising all other infection sources, including abdominal and urinary sources independently associated with lower MRSA risk, pulmonary infection appears relatively high-risk. Once these sources are included as covariates, the apparent association is no longer observed. This finding is consistent with those of prior studies that similarly did not identify pulmonary or respiratory infection as an independent MRSA risk factor [4]. Overall, these findings suggest that accompanying exposure-related factors, rather than pulmonary infection itself, underlie the increased risk of MRSA.

While CRP is a nonspecific inflammatory marker, studies report higher levels in patients with MRSA infections [23]. In a pediatric study of acute osteomyelitis, a CRP level > 73.23 µg/mL (7.32 mg/dL) correlated with increased MRSA risk, while persistent CRP elevation after 3 days of therapy is associated with treatment failure, often requiring antibiotic modification [24]. Although derived from a pediatric population, the CRP threshold is consistent with our identified cutoff (≥6 mg/dL).

These findings question the utility of traditional MRSA risk factors in ED settings. Hemodialysis, immunosuppression, and recent antibiotic use were not independently associated with MRSA in this acutely ill, heterogeneous population. This may reflect differences in patient characteristics, microbial epidemiology, and ED care urgency. These observations align with updated Surviving Sepsis Campaign and Infectious Diseases Society of America/American Thoracic Society pneumonia guidelines, which emphasize individualized therapy based on local epidemiology rather than historical healthcare-associated pneumonia criteria [1,4,25].

Given the low overall MRSA prevalence and limited value of traditional markers, infection source, CRP level, and recent hospitalization may nonetheless serve as practical, readily available clinical factors when assessing the likelihood of MRSA in the ED. Recent European studies of simplified MRSA risk scores show greater specificity and cost-effectiveness than those of broader screening strategies based on complex criteria [26]. These clinical factors should be viewed as pre-microbiological risk indicators rather than as a stand-alone decision rule, informing the initial probability of MRSA while awaiting microbiological confirmation and enabling prompt de-escalation once rapid diagnostic or susceptibility results are available. Although our study was not designed to develop or validate a treatment algorithm, the observed heterogeneity in associations of MRSA with infection source and healthcare exposure is conceptually consistent with the broader shift toward individualized, risk-stratified antimicrobial decision-making [27]. Therefore, future studies with external validation across diverse geographic and healthcare settings are warranted before these factors can be used as independent prescribing criteria.

### Limitations

This study has some limitations. First, the registry lacked data on previous MRSA colonization or infection, limiting assessment of this established risk factor. However, this reflects a common challenge in ED practice, where this information is often unavailable [28]. Second, MRSA classification relied on assessments made by treating physicians without centralized adjudication. Thus, isolates from nonsterile specimens, particularly sputum, may have represented colonization. Routine MRSA surveillance and specimen-quality data were unavailable. However, sensitivity analysis restricted to MRSA bacteremia yielded consistent findings, supporting the robustness of the primary results (Supplementary Table S1). Third, all participating centers were tertiary or university-affiliated hospitals in South Korea; therefore, the prevalence and associated factors observed in this cohort may not be generalizable to community hospitals, non-tertiary settings, or regions with different MRSA epidemiology and diagnostic practices. Fourth, the retrospective analysis of a prospective registry precludes causal inference, and data on antibiotic timing or appropriateness were not collected. Fifth, the adjusted analysis included complete cases and excluded 10.4% of the cohort (1189 of 11,477 patients) because of missing data for at least one covariate, predominantly CRP (missing in 1130 patients, 9.8%); missingness in the remaining covariates was minimal (≤0.3%). Although most patients had complete covariate data, excluded patients may have differed systematically from included patients, potentially introducing selection bias and reducing statistical precision. However, sensitivity analysis using multiple imputation for missing covariate data yielded results consistent with the complete-case analysis (Supplementary Table S4). Finally, the relatively low MRSA prevalence may have limited statistical power to detect associations with less common risk factors. This was particularly relevant for catheter-related infection, which was based on few MRSA cases; sensitivity analysis using Firth’s penalized-likelihood logistic regression yielded a similar estimate (OR = 6.322; 95% CI, 2.147–18.613), supporting the robustness of the association despite the limited number of events.

## 4. Materials and Methods

This study was conducted and reported according to the Strengthening the Reporting of Observational Studies in Epidemiology (STROBE) guidelines.

### 4.1. Study Design, Population, and Data Collection

This cohort study was a secondary analysis of an ongoing nationwide, multicenter, prospective observational cohort study. For this analysis, we used data collected between 1 September 2019, and 31 December 2022, from a sepsis registry established by the Korean Sepsis Alliance that includes 16 tertiary or university-affiliated hospitals across South Korea. During the study period, all patients presenting to participating centers were consecutively screened for eligibility. Detailed enrollment procedures and data collection have been described previously [29].

Adult patients (aged ≥19 years) diagnosed with sepsis according to the Third International Consensus Definitions for Sepsis and Septic Shock were included and followed until hospital discharge or mortality. Patients with sepsis presenting to the ED were included regardless of admission to a general ward or ICU. Institutional review boards at each participating center approved the study protocol, and informed consent was waived because of the observational study design. Patient data were anonymized and de-identified to ensure confidentiality. Blood cultures were obtained from all patients according to the Korean Sepsis Alliance registry protocol. MRSA infection was defined as microbiological identification of MRSA in a clinical specimen, including blood, urine, sputum, pus, tissue, or sterile fluids such as pleural, peritoneal, or cerebrospinal fluid, when the organism was considered the causative pathogen of the index sepsis episode according to the predefined registry definitions. However, standardized specimen-specific microbiological criteria and centralized microbiological adjudication were not available. Physiologic variables and inflammatory markers were recorded during the initial evaluation of the index sepsis episode, whereas final culture results and confirmation of the infection source could become available after ED disposition; infections developing after admission to the participating hospital were not included as study outcomes. Since infection-source classification could incorporate findings obtained after ED disposition, particularly culture results used to classify catheter-related and urinary sources, these categories may be subject to incorporation bias when interpreted as available at initial presentation. Data on infection sources and causative pathogens, including MRSA, were collected by trained site coordinators through structured medical-record review using a standardized case report form and centrally reviewed for completeness and logical consistency at the coordinating center, as previously described [24]. For patients with MRSA-positive cultures, culture sources were ascertained from structured culture-source data and free-text microbiology documentation; multiple specimen types could be recorded per patient. Patients with and without MRSA were compared (MRSA and non-MRSA groups, respectively).

Collected variables included demographic characteristics (age, sex, and body mass index); health status (Charlson Comorbidity Index, Clinical Frailty Scale, and comorbidities); recent healthcare exposures (hospitalization for ≥2 days within the previous 90 days; antibiotic use, dialysis, or wound care within the previous 30 days); admission details (transfer from another hospital, residence in a skilled nursing facility, or arrival from home); and current immunosuppressive medication use. Illness severity was evaluated using the SOFA score and initial physiologic and laboratory parameters obtained at ED presentation. Infection-related variables included the infection source (respiratory tract, abdomen, urinary tract, SSTI, catheter-related, neurologic, or unclear primary site) and identified pathogens, when available. The infection source was unrecorded for 935 patients (8.1%; 28 [14.8%] in the MRSA group and 907 [8.0%] in the non-MRSA group). Catheter-related infection was defined as bacteremia in a patient with an intravascular device and no other identifiable bloodstream infection source.

### 4.2. Statistical Analysis

Descriptive statistics were used to summarize baseline characteristics. Continuous variables were expressed as means (standard deviations) or medians (interquartile ranges), according to data distribution, and compared using Student’s *t*-test or the Mann–Whitney U test, as appropriate. Categorical variables were reported as frequencies and percentages and compared using the chi-square or Fisher’s exact test, as appropriate. Receiver operating characteristic curves were generated to assess the predictive performance of CRP for MRSA, and the optimal cutoff value was determined by maximizing the Youden index. Since the threshold was derived and evaluated in the same dataset, internal validation was performed using 1000 bootstrap resamples, with cutoff selection repeated within each resample, and optimism-corrected estimates of AUROC, sensitivity, specificity, PPV, and NPV calculated. Variables with a *p* value < 0.10 in univariable analyses were considered candidate predictors for multivariable logistic regression. Candidate variables were evaluated for clinical relevance and potential multicollinearity, and those representing overlapping clinical concepts were excluded. Multicollinearity was assessed using variance inflation factors (VIFs), with VIF ≥ 5 considered potentially problematic; all covariates in the final model had VIFs below this threshold (maximum, 1.94). Multivariable analyses were performed using complete-case analysis, excluding observations with missing values in variables included in the final model. Results were reported as ORs with corresponding 95% CIs. A two-sided *p* value < 0.05 was considered statistically significant. Statistical analyses were performed using R software (version 4.4.2; R Foundation for Statistical Computing, Vienna, Austria).

To assess the robustness of the primary findings, we performed four sensitivity analyses: restricting the analysis to patients with MRSA bacteremia (*n* = 96); restricting it to the culture-positive cohort (*n* = 6606) to compare MRSA with microbiologically confirmed non-MRSA infection; using mixed-effects logistic regression with a random intercept for participating center to account for clustering across 16 hospitals, although this adjustment could not fully capture between-center differences in local microbiology, culture practices, or antimicrobial policies; and using multiple imputation by chained equations (m = 20 imputed datasets) to address missing covariate data. Results were consistent with the primary analysis across all four approaches and are presented in Supplementary Tables S1–S4, respectively.

Since the association between catheter-related infection and MRSA was based on few events, a sensitivity analysis was performed using Firth’s penalized likelihood logistic regression with the same set of covariates as the primary model to address potential sparse-data bias.

## 5. Conclusions

In this large multicenter cohort of patients with sepsis presenting to the ED, MRSA infection was uncommon. Recent hospitalization, elevated CRP, and catheter-related infections were independently associated with MRSA, whereas abdominal infections and UTIs were associated with lower odds of MRSA infection. These findings suggest that empiric MRSA coverage may not be routinely required for patients with abdominal or urinary sources of sepsis without specific MRSA risk factors, thereby supporting antimicrobial stewardship in the ED.

## Figures and Tables

**Table 1:** Baseline characteristics of patients with sepsis stratified based on MRSA infection status.

Characteristics	All (*n* = 11,477)	MRSA ^1^ Group (*n* = 189)	Non-MRSA Group (*n* = 11,288)	*p*-Value
**General characteristics**				
Age, years	73.2 (13.5)	75.9 (12.9)	73.1 (13.5)	0.005
Body mass index, kg/m^2^	21.6 (4.3)	21.6 (4.6)	21.6 (4.2)	0.900
Male sex	6381 (55.6)	116 (61.4)	6265 (55.5)	0.124
Clinical Frailty Scale	6 [4–7]	7 [5–8]	6 [4–7]	<0.001
Charlson Comorbidity Index	6 [5–8]	7 [5–8]	6 [5–8]	0.168
Initial SOFA ^2^ score	6 [4–8]	7 [5–9]	6 [4–8]	0.001
Septic shock	1654 (14.4)	30 (15.9)	1624 (14.4)	0.637
**Comorbidity**				
Diabetes mellitus	3949 (34.4)	73 (38.6)	3876 (34.3)	0.249
Chronic lung disease	1401 (12.2)	25 (13.2)	1376 (12.2)	0.749
Cardiovascular disease	2161 (18.8)	43 (22.8)	2118 (18.8)	0.195
Chronic kidney disease ^3^	1185 (10.3)	32 (16.9)	1153 (10.2)	0.004
Chronic neurologic disease	4501 (39.2)	101 (53.4)	4400 (39.0)	<0.001
Chronic liver disease	848 (7.4)	10 (5.3)	838 (7.4)	0.331
Connective tissue disease	268 (2.3)	5 (2.6)	263 (2.3)	0.966
Solid malignant tumor	4015 (35.0)	53 (28.0)	3962 (35.1)	0.052
Hematologic malignancy	467 (4.1)	4 (2.1)	463 (4.1)	0.236
**Immunosuppressi** **on**				
Acquired immunodeficiency syndrome	13 (0.1)	1 (0.5)	12 (0.1)	0.533
Immunosuppressant use	271 (2.4)	4 (2.1)	267 (2.4)	1.000

Bold text indicates category headings within the table. Values are presented as mean (SD), median [IQR], or number (%). SD, standard deviation; IQR, interquartile range. ^1^ MRSA, methicillin-resistant *Staphylococcus aureus*. ^2^ SOFA, Sequential Organ Failure Assessment. ^3^ Chronic kidney disease is defined as kidney damage or a glomerular filtration rate < 60 mL/min/1.73 m^2^ for 3 months.

**Table 2:** Infection characteristics and healthcare exposure of patients with sepsis stratified based on MRSA infection status.

Characteristics	All (*n* = 11,477)	MRSA ^1^ Group (*n* = 189)	Non-MRSA Group (*n* = 11,288)	*p*-Value
**Source of infection**				
Pulmonary	4774 (41.6)	103 (54.5)	4671 (41.4)	<0.001
Abdominal	2517 (21.9)	9 (4.8)	2508 (22.2)	<0.001
Urinary	1800 (15.7)	16 (8.5)	1784 (15.8)	0.008
Skin and soft tissue	263 (2.3)	10 (5.3)	253 (2.2)	0.011
Catheter-related	34 (0.3)	5 (2.6)	29 (0.3)	<0.001
Neurologic	72 (0.6)	1 (0.5)	71 (0.6)	1.000
Unclear primary site	1082 (9.4)	17 (9.0)	1065 (9.4)	0.936
Source of infection not recorded	935 (8.1)	28 (14.8)	907 (8.0)	0.001
**Infection type**				
Community-acquired	7962 (69.4)	89 (47.1)	7873 (69.7)	<0.001
Nursing home-acquired	1193 (10.4)	33 (17.5)	1160 (10.3)	0.002
Nursing hospital-acquired	1667 (14.5)	54 (28.6)	1613 (14.3)	<0.001
Hospital-acquired	655 (5.7)	13 (6.9)	642 (5.7)	0.588
Antimicrobial history (past 30 days)	3578 (31.2)	70 (37.0)	3508 (31.1)	0.094
Hospitalization history (past 90 days)	5178 (45.1)	114 (60.3)	5064 (44.9)	<0.001
Dialysis history (past 30 days)	403 (3.5)	12 (6.3)	391 (3.5)	0.053
Recent wound management (past 30 days)	315 (2.7)	9 (4.8)	306 (2.7)	0.137

Bold text indicates category headings within the table. Values are presented as mean (SD), median [IQR], or number (%). SD, standard deviation; IQR, interquartile range. ^1^ MRSA, methicillin-resistant *Staphylococcus aureus*.

**Table 3:** Laboratory findings of patients with and without MRSA.

Variables	All (*n* = 11,477)	MRSA ^1^ Group (*n* = 189)	Non-MRSA Group (*n* = 11,288)	*p*-Value
White blood cell count, ×10^3^/µL	13.2 (11.0)	13.7 (8.5)	13.2 (11.0)	0.517
Neutropenia	951 (8.3)	10 (5.3)	941 (8.3)	0.170
Hemoglobin, g/dL	10.9 (2.6)	10.3 (2.5)	10.9 (2.6)	0.003
Platelet count, ×10^3^/µL	192.8 (120.8)	211.9 (134.5)	192.5 (120.5)	0.028
Blood urea nitrogen, mg/dL	39.9 (29.5)	46.9 (35.9)	39.8 (29.3)	0.001
Creatinine, mg/dL	1.9 (1.8)	1.9 (1.8)	1.9 (1.7)	0.870
Total bilirubin, mg/dL	1.6 (3.0)	1.4 (3.7)	1.6 (3.0)	0.339
Prothrombin time, INR ^2^	1.4 (2.3)	1.3 (0.5)	1.4 (2.3)	0.737
Albumin, g/dL	2.9 (0.7)	2.7 (0.7)	2.9 (0.7)	<0.001
AST ^3^, IU/L	120.7 (533.4)	71.8 (171.9)	121.5 (537.3)	0.204
ALT ^4^, IU/L	62.7 (210.9)	42.6 (93.3)	63.1 (212.3)	0.192
C-reactive protein, mg/dL	13.2 (10.4)	15.9 (10.3)	13.1 (10.4)	<0.001
Procalcitonin, ng/mL	18.6 (45.9)	15.2 (39.3)	18.6 (46.0)	0.396
Lactate, mmol/L	3.9 (3.4)	3.7 (3.4)	3.9 (3.4)	0.556
Sodium, mmol/L	136.0 (8.6)	137.3 (9.9)	136.0 (8.6)	0.056
Potassium, mmol/L	4.3 (1.0)	4.3 (1.0)	4.3 (1.0)	0.984
Chloride, mmol/L	101.8 (9.0)	103.3 (10.4)	101.8 (9.0)	0.021
Arterial pH	7.3 (0.1)	7.3 (0.13)	7.3 (0.13)	0.830
PaO_2_, mmHg	95.6 (53.8)	102.1 (66.4)	95.5 (53.5)	0.105
PaCO_2_, mmHg	33.4 (13.7)	35.0 (12.3)	33.4 (13.7)	0.122

Values are presented as mean (SD) or number (%). SD, standard deviation. ^1^ MRSA, methicillin-resistant *Staphylococcus aureus*. ^2^ INR, international normalized ratio. ^3^ AST, aspartate aminotransferase. ^4^ ALT, alanine aminotransferase.

**Table 4:** Multivariable logistic regression for factors associated with MRSA **^1^** in patients with sepsis.

Variables	OR ^2^	95% CI ^3^	*p*-Value
Age	1.006	0.992–1.020	0.397
Clinical Frailty Scale	1.041	0.945–1.147	0.410
Initial SOFA ^4^ score	1.034	0.973–1.100	0.282
Chronic kidney disease	1.253	0.744–2.111	0.397
Solid malignant tumor	0.772	0.530–1.125	0.178
Chronic neurologic disease	1.407	0.980–2.019	0.064
Pulmonary	0.989	0.671–1.457	0.955
Abdominal	0.199	0.092–0.430	<0.001
Urinary	0.407	0.215–0.773	0.006
Skin and soft tissue	1.983	0.932–4.220	0.075
Catheter-related	5.988	1.939–18.491	0.002
Antimicrobial history (past 30 days)	1.012	0.692–1.479	0.952
Hospitalization history (past 90 days)	1.655	1.144–2.393	0.007
Dialysis history (past 30 days)	1.030	0.462–2.299	0.942
Hemoglobin	0.958	0.893–1.028	0.230
Platelet count	1.001	1.000–1.002	0.054
Blood urea nitrogen	1.004	0.999–1.009	0.117
Albumin	0.771	0.582–1.022	0.071
C-reactive protein (≥6 mg/dL)	2.187	1.413–3.386	<0.001

^1^ MRSA, methicillin-resistant *Staphylococcus aureus*. ^2^ OR, odds ratio. ^3^ CI, confidence interval. ^4^ SOFA, Sequential Organ Failure Assessment.

## Data Availability

The data presented in this study are available on request from the corresponding author due to privacy and ethical restrictions.

## Supplementary Materials

The following supporting information can be downloaded at https://www.mdpi.com/article/10.3390/antibiotics15090915/s1, Table S1. Sensitivity analysis restricted to blood culture-confirmed MRSA bacteremia: multivariable logistic regression for factors associated with MRSA bacteremia in patients with sepsis. Table S2. Sensitivity analysis restricted to the culture-positive cohort: multivariable logistic regression for factors associated with MRSA bacteremia. Table S3. Sensitivity analysis accounting for center-level clustering: mixed-effects logistic regression for factors associated with MRSA. Table S4. Sensitivity analysis for missing covariate data: multiple imputation for factors associated with MRSA.

## Abbreviations

MRSA	Methicillin-resistant *Staphylococcus aureus*
ICU	Intensive care unit
SSTIs	Skin and soft tissue infections
ED	Emergency department
SOFA	Sequential Organ Failure Assessment
CRP	C-reactive protein
OR	Odds ratio
CI	Confidence interval
UTI	Urinary tract infection
PPV	Positive predictive value
NPV	Negative predictive value
AUROC	Area under the receiver operating characteristic curve
VIF	Variance inflation factor
