# Supplementary material for: Factors Associated with Methicillin-Resistant Staphylococcus aureus Infection Among Patients with Sepsis Presenting to Emergency Departments: A Nationwide Multicenter Cohort Study
Source: Antibiotics (Basel). 2026 Sep 16;15(9):915. doi: 10.3390/antibiotics15090915 (PMC13603083; doi:10.3390/antibiotics15090915)
Supplement: Supplementary file 1 [file antibiotics-15-00915-s001.zip › Supplement Table S1.pdf]

Table S1. Sensitivity Analysis Restricted to Blood Culture-Confirmed MRSA Bacteremia: Multivariable Logistic Regression for Predictors of MRSA Bacteremia in Patients With Sepsis

Variables	OR[a]	95% CI[b]	*p*-value
Age	1.016	0.998-1.035	0.084
Source of Infection			
Abdominal	0.171	0.062 – 0.470	<0.001
Urinary	0.240	0.087 – 0.661	0.006
Catheter-related	11.075	3.619 – 33.892	<0.001
C-reactive protein (≥ 6 mg/dL)	4.312	2.071-8.979	<0.001
Hospitalization history in the past 90 days	1.784	1.132-2.814	0.013
Dialysis history in past 30 days	2.095	0.934-4.702	0.073

[a]OR: Odds Ratio

[b]CI: Confidence Interval

Cases were patients with blood culture-confirmed MRSA bacteremia; non-MRSA patients served as the comparison group. Because of the limited number of bacteremia events, a prespecified parsimonious model was used, restricted to covariates from the primary analysis (infection source: abdominal, urinary, and catheter-related; hospitalization within the past 90 days; dialysis within the past 30 days; CRP ≥ 6 mg/dL) plus age, selected before bacteremia-specific results were examined (complete-case n = 10,261; MRSA bacteremia events = 81; events-per-variable = 11.6).
