# Supplementary material for: Factors Associated with Methicillin-Resistant Staphylococcus aureus Infection Among Patients with Sepsis Presenting to Emergency Departments: A Nationwide Multicenter Cohort Study
Source: Antibiotics (Basel). 2026 Sep 16;15(9):915. doi: 10.3390/antibiotics15090915 (PMC13603083; doi:10.3390/antibiotics15090915)
Supplement: Supplementary file 1 [file antibiotics-15-00915-s001.zip › Supplement Table S2.pdf]

Table S2. Sensitivity Analysis Restricted to the Culture-Positive Cohort: Multivariable Logistic Regression for Factors Associated With MRSA[a] bacteremia

Variables	OR[b]	95% CI[c]	*p*-value
Age	1.004	0.990-1.018	0.573
Clinical Frailty Scale	1.005	0.910-1.110	0.925
Initial SOFA[d] score	1.018	0.957-1.083	0.567
Chronic kidney disease	1.357	0.793-2.321	0.265
Solid malignant tumor	0.912	0.622-1.336	0.635
Chronic neurologic disease	1.358	0.941-1.960	0.102
Source of Infection			
Pulmonary	1.207	0.816 – 1.784	0.347
Abdominal	0.185	0.085 – 0.403	<0.001**
Urinary	0.301	0.158 – 0.572	<0.001**
Skin and soft tissue	1.800	0.833 – 3.891	0.135
Catheter-related	4.241	1.361 – 13.212	0.013**
Antimicrobial history in the past 30 days	1.122	0.762-1.652	0.560
Hospitalization history in the past 90 days	1.625	1.117-2.365	0.011**
Dialysis history in past 30 days	1.222	0.538-2.775	0.631
Hemoglobin	0.951	0.884-1.023	0.175
Platelet count	1.002	1.000-1.003	0.009**
Blood urea nitrogen	1.004	0.998-1.009	0.181
Albumin	0.820	0.614-1.093	0.176
C-reactive protein (≥ 6 mg/dL)	1.867	1.202-2.901	0.005**

[a]MRSA, methicillin-resistant *Staphylococcus aureus*

[b]CI: Confidence Interval

[c]OR: Odds Ratio

[d]SOFA, Sequential Organ Failure Assessment

Cohort restricted to patients with an identified causative pathogen (n = 6,606); MRSA (n = 189) was compared with microbiologically confirmed non-MRSA causative pathogens (n = 6,417) using the same 19-covariate multivariable model as the primary analysis (complete-case n = 5,972; MRSA events = 166; events-per-variable = 8.7).
