# Supplementary material for: Factors Associated with Methicillin-Resistant Staphylococcus aureus Infection Among Patients with Sepsis Presenting to Emergency Departments: A Nationwide Multicenter Cohort Study
Source: Antibiotics (Basel). 2026 Sep 16;15(9):915. doi: 10.3390/antibiotics15090915 (PMC13603083; doi:10.3390/antibiotics15090915)
Supplement: Supplementary file 1 [file antibiotics-15-00915-s001.zip › Supplement Table S3.pdf]

Table S3. Sensitivity Analysis Accounting for Center-Level Clustering: Mixed-Effects Logistic Regression for Factors Associated With MRSA[a]

Variables	OR[b]	95% CI[c]	*p*-value
Age	1.003	0.990–1.017	0.639
Clinical Frailty Scale	1.018	0.919–1.128	0.737
Initial SOFA[d] score	1.017	0.956–1.082	0.600
Chronic kidney disease	1.256	0.740–2.131	0.399
Solid malignant tumor	0.844	0.575–1.239	0.386
Chronic neurologic disease	1.429	0.991–2.060	0.056
Source of Infection			
Pulmonary	0.992	0.669–1.472	0.970
Abdominal	0.207	0.095–0.451	<0.001**
Urinary	0.387	0.202–0.741	0.004**
Skin and soft tissue	1.988	0.925–4.274	0.079
Catheter-related	6.129	1.915–19.620	0.002**
Antimicrobial history in the past 30 days	1.085	0.740–1.591	0.676
Hospitalization history in the past 90 days	1.648	1.138–2.387	0.008**
Dialysis history in past 30 days	1.101	0.488–2.485	0.816
Hemoglobin	0.963	0.897–1.034	0.296
Platelet count	1.001	1.000–1.002	0.096
Blood urea nitrogen	1.005	1.000–1.010	0.075
Albumin	0.667	0.494–0.901	0.008**
C-reactive protein (≥ 6 mg/dL)	2.097	1.347–3.263	0.001**

[a]OR: Odds Ratio

[b]CI: Confidence Interval

Sensitivity analysis using mixed-effects logistic regression with a random intercept for participating center, applying the same 19-covariate multivariable model as the primary analysis (complete-case n = 10,288; MRSA events = 166). Estimated center-level (random-intercept) variance = 0.221 (SD = 0.470); intraclass correlation coefficient (ICC) = 0.063.
