# Supplementary material for: Factors Associated with Methicillin-Resistant Staphylococcus aureus Infection Among Patients with Sepsis Presenting to Emergency Departments: A Nationwide Multicenter Cohort Study
Source: Antibiotics (Basel). 2026 Sep 16;15(9):915. doi: 10.3390/antibiotics15090915 (PMC13603083; doi:10.3390/antibiotics15090915)
Supplement: Supplementary file 1 [file antibiotics-15-00915-s001.zip › Supplement Table S4.pdf]

Table S4. Sensitivity Analysis for Missing Covariate Data: Multiple Imputation for Factors Associated With MRSA[a]

Variables	OR[b]	95% CI[c]	p-value
Age	1.008	0.995–1.021	0.229
Clinical Frailty Scale	1.022	0.934–1.117	0.639
Initial SOFA[d] score	1.039	0.981–1.100	0.188
Chronic kidney disease	1.392	0.860–2.254	0.178
Solid malignant tumor	0.762	0.535–1.085	0.132
Chronic neurologic disease	1.437	1.025–2.015	0.036**
Pulmonary	0.910	0.635–1.305	0.609
Abdominal	0.184	0.089–0.380	<0.001**
Urinary	0.377	0.211–0.674	<0.001**
Skin and soft tissue	1.784	0.875–3.638	0.111
Catheter-related	6.504	2.337–18.102	<0.001**
Antimicrobial history (past 30 days)	1.088	0.765–1.546	0.640
Hospitalization history (past 90 days)	1.653	1.170–2.336	0.004**
Dialysis history (past 30 days)	1.018	0.485–2.134	0.963
Hemoglobin	0.978	0.915–1.044	0.499
Platelet count	1.001	1.000–1.003	0.017**
Blood urea nitrogen	1.004	0.999–1.008	0.137
Albumin	0.726	0.556–0.948	0.019**
C-reactive protein (≥ 6 mg/dL)	2.077	1.337–3.227	0.001**

[a]MRSA, methicillin-resistant Staphylococcus aureus
[b]OR: odds ratio
[c]CI: confidence interval
[d]SOFA: Sequential Organ Failure Assessment.
Multiple imputation performed using chained equations (mice, R package; m = 20 imputed datasets, 20 iterations, predictive mean matching), including the outcome (MRSA) and all model covariates. Continuous C-reactive protein (CRP) was imputed and CRP ≥ 6 mg/dL was subsequently derived in each imputed dataset (missingness in CRP accounted for the great majority of excluded complete cases: 1,130/11,477 patients, 9.8%). Estimates were pooled across imputations using Rubin's rules. Fraction of missing information was 15.2% for CRP (≥ 6 mg/dL) and ≤2% for all other covariates.
